# A Narrative Review of Cutaneous Wound-Healing Botanicals in South Africa: Integrating Ethnomedicinal Use, Pharmacological Evidence, and Conservation Prioritization

**DOI:** 10.3390/ph19101587

**Published:** 2026-10-08

**Authors:** Siboniso Sithole, Carl Mateta, Sphamandla Hlatshwayo, Sanele Mhlungu, Nokukhanya Thembane, Phumzile Afrika, Sibusiso Senzani, Exnevia Gomo, Nceba Gqaleni, Mlungisi Ngcobo

**Affiliations:** 1 Sub-Discipline of Traditional Medicine, School of Medicine, College of Health Sciences, University of KwaZulu-Natal, Durban 4041, South Africa209504114@stu.ukzn.ac.za (S.M.);; 2 National Institute of Health Research, Ministry of Health and Child Care, Harare P.O. Box CY 1122, Zimbabwe; 3 Department of Biomedical Sciences, Faculty of Applied and Health Sciences, Mangosuthu University of Technology, Durban 4026, South Africa; 4 Discipline of Medical Microbiology, School of Medicine, College of Health Sciences, University of KwaZulu-Natal, Durban 4000, South Africa; 5 Medical Laboratory Sciences Unit, Faculty of Medicine and Health Sciences, University of Zimbabwe, Mount Pleasant, Harare P.O. Box MP 167, Zimbabwe; 6 Africa Health Research Institute, Nelson R. Mandela School of Medicine, 3rd Floor K-RITH Tower Building, Durban 4001, South Africa

**Keywords:** ethnobotany, ethnopharmacology, conservation status, medicinal plants, traditional medicine, wound healing, South Africa

## Abstract

African Traditional Medicine (ATM) remains an important component of primary healthcare in South Africa; however, evidence for plants used in cutaneous wound care is dispersed across ethnobotanical, pharmacological, toxicological, and conservation literature. This narrative review integrates these evidence streams and proposes a conservation- and safety-aware translational prioritization framework for wound-healing botanicals used in South African ethnomedical practice. A structured search of PubMed, ScienceDirect, Web of Science, Google Scholar, and SciFinder identified eligible peer-reviewed ethnobotanical and preclinical studies published between January 1999 and June 2026. After taxonomic harmonization, 173 accepted taxa were documented from South African ethnobotanical reports as being traditionally used for wound care. Only seven taxa had direct wound closure evidence in relevant in vitro or in vivo models, while twenty-eight taxa had wound-relevant anti-inflammatory evidence. Reported mechanisms included keratinocyte and fibroblast migration, collagen-associated responses, angiogenesis, wound contraction, and inflammatory modulation. The evidence base remains uneven: many taxa are supported primarily by traditional use, while dose, formulation, treatment frequency, duration of use, dermal safety, and sustainable sourcing are poorly reported. Safety concerns were identified for selected toxic or irritant taxa, and destructive harvesting of roots, bulbs, corms, rhizomes, and bark may increase conservation risk. Overall, the review highlights a substantial gap between ethnomedical documentation and pharmacological validation, and it provides a prioritization strategy to guide future phytochemical, mechanistic, formulation, dermal-safety, and preclinical wound-healing research in South Africa.

## 1. Introduction

A wound is defined as the disruption or damage to skin tissue function due to various conditions such as trauma, burns, microbial infection, and surgery [1]. Based on the underlying cause, wounds can be categorized as open (exposed to the environment) or closed (not exposed to the environment). Based on healing physiology, wounds are further classified as acute or chronic depending on the time required for repair. Acute wounds progress through the normal phases of healing in an orderly and timely manner and typically achieve functional closure within approximately 2–4 weeks, depending on wound characteristics and patient factors [2]. However, tissue remodeling and scar maturation may continue for several months after wound closure. In contrast, chronic wounds fail to progress through the normal healing sequence and remain unhealed beyond the expected period of repair, often being defined as wounds that persist for approximately 3 months or longer [3]. The incidence of acute and chronic wounds is increasing globally, imposing a substantial financial burden on patients and healthcare systems [4]. This trend is driven in part by the increasing prevalence of comorbidities such as obesity, peripheral vascular disease, diabetes, and venous hypertension as the population ages [5].

Global statistics indicate that approximately 40 million patients are affected by chronic skin wounds worldwide [6]. The prevalence of acute and chronic wounds in South Africa remains poorly documented [7,8]. However, recent reports indicate that around 1.6 million South Africans experience burn wounds each year. Wound treatment poses social, clinical, and economic challenges. The annual cost of wound care in South Africa remains incompletely characterized; however, global expenditure estimates suggest that approximately USD 28.1 billion to USD 96.8 billion is spent on the treatment of acute and chronic wounds [9]. One published estimate placed the 2019 cost of wound care in South Africa at approximately USD 1.163 billion [10]. The burden of chronic wounds is particularly high in older adults, with global prevalence estimates of 1.67 chronic wounds per 1000 people [11].

Treatment of skin wounds is a serious global health challenge [12]. This is partly attributable to inadequate wound-management systems and the high risk of microbial infection. Staphylococcus aureus, Pseudomonas aeruginosa, and Streptococcus spp. are among the bacteria that commonly colonize wounds within 3 days of injury and contribute to infection [13]. These organisms can form biofilms at the wound site, thereby delaying healing and promoting chronicity [14,15]. Persistent bacterial infection and prolonged inflammation can inhibit orderly wound repair and delay healing [16].

Conventional treatment includes wound dressings that protect the epidermal layer of the skin, together with agents that promote healing and synthetic antibiotics. Treatment strategies that support oxygenation, tissue perfusion, and a moist wound-healing environment are required to accelerate the phases of repair [17]. Despite improvements in wound management, antimicrobial resistance remains a major challenge, and many patients remain unresponsive to current treatment [18]. Current dressings, hydrogels, topical agents, and remedies are often inadequate for chronic wounds. Accordingly, alternative wound-healing therapies that could reduce cost and mitigate antimicrobial resistance merit further investigation [19].

African Traditional Medicine represents a locally relevant source of potential wound-care agents. Chemically diverse plant extracts may exert complementary antimicrobial, antibiofilm, anti-inflammatory, antioxidant, and tissue-repair activities, potentially providing multi-target effects that differ from the more defined molecular targets of many conventional antibiotics [20]. Such multi-target activity may be particularly relevant to complex or infected wounds and could support the development of adjunctive therapies against infections involving antimicrobial-resistant organisms [21]. Furthermore, the local availability of medicinal plants may facilitate the development of context-appropriate and potentially affordable wound-care interventions. However, these potential benefits require rigorous pharmacological validation, antimicrobial resistance and antibiofilm studies, chemical standardization, dermal-safety assessment, sustainable sourcing, and cost-effectiveness evaluation.

The World Health Organization (WHO) previously indicated that around 80% of the population in developing regions use traditional medicines as their primary source of healthcare [22]. More than 27 million individuals in South Africa depend on traditional medicine for healthcare [23]. In South Africa, around 70% of Indigenous people consult traditional health practitioners for primary healthcare needs. Many medicinal plants in South Africa have been used traditionally for centuries to promote wound healing, yet limited scientific evidence exists for their efficacy [24]. Their continued use is largely attributed to cost-effectiveness, accessibility, and the perception that they have fewer side effects. The high cost of Western medicine has also contributed to sustained demand for plant-based remedies [25]. It has been estimated that approximately 3000 plant species are used by traditional healers for the treatment of diseases in South Africa [26]. Currently, there are no comprehensive reviews that integrate ethnobotanical, ethnopharmacological, safety, and conservation evidence for medicinal plants used for wound healing in the South African context.

Previous reviews on selected southern African medicinal plants indicate that some taxa may support wound repair through collagen synthesis, re-epithelialization, and wound contraction, and that phytochemical classes such as flavonoids, triterpenes, and alkaloids may contribute to wound-relevant bioactivity [27]. However, the current state of the field remains fragmented: ethnobotanical documentation is extensive, direct wound closure validation is limited, toxicological evidence is uneven, and conservation risk is rarely integrated into pharmacological prioritization. An updated South Africa-specific synthesis is therefore warranted. This review aims to integrate ethnomedicinal use, pharmacological evidence, safety considerations, conservation status, and translational prioritization for cutaneous wound-healing botanicals documented in South African ethnomedical practice.

## 2. Results

### 2.1. Ethnobotanical Survey

A total of 1892 records were identified through database searches. After the removal of 304 duplicate records, 1587 records underwent title and abstract screening. Of these, 947 records were excluded because they did not meet the preliminary eligibility criteria, and 640 reports were sought for retrieval. However, 599 reports could not be retrieved, leaving 41 full-text reports for eligibility assessment. Following full-text assessment, 18 reports were excluded because they did not meet the predefined inclusion criteria. Consequently, 23 articles were included in the final narrative synthesis.

After taxonomic harmonization of currently accepted names and synonyms, 173 accepted medicinal plant taxa were documented as being used traditionally for wound healing in South Africa (Table 1). Ethnobotanical documentation was geographically uneven, with the strongest representation in Limpopo, the Eastern Cape, and KwaZulu-Natal, indicating broader coverage of these provinces than of several other parts of the country. Knowledge on wound-care plants was documented from Indigenous communities, traditional healers, herbalists, and other local knowledge holders, and most preparations were applied topically as poultices, dressings, pastes, lotions, decoctions, washes, or ash-based applications. As the scope of this review was defined by medicinal plant use in South African wound-care practice rather than by floristic origin, taxa reported in South African ethnobotanical studies were retained irrespective of whether the species were indigenous, introduced, cultivated, or naturalized. Nevertheless, the majority of ethnobotanical sources lacked detailed information on dose, treatment frequency, duration of application, and formulation standardization, which restricts reproducibility and limits the translational value of these reports. Plant-part analysis showed that leaves were the dominant component in traditional wound-care preparations, followed by roots and whole plants. In some cases, plant material was first burned to ash before being applied topically to open wounds or mixed with porridge as an oral preparation. As shown in Figure 1, the 173 medicinal plant taxa traditionally used for wound healing in South Africa belong to several botanical families, with varying frequencies of reported plant parts used in traditional wound healing practices.

### 2.2. Ethnopharmacological Evidence of Wound Closure and Anti-Inflammatory Activities

#### 2.2.1. In Vitro and In Vivo Wound Closure Studies

Of the 173 accepted taxa identified after taxonomic harmonization, only seven (4.0%) had wound-relevant experimental evidence in in vitro or in vivo models (Table 2), while 166 taxa (96.0%) lacked such validation within the predefined scope. This disparity demonstrates the substantial gap between South Africa’s ethnobotanical inventory and experimental wound-healing research [51,52,53]. Among the evaluated taxa, *B. frutescens* had the strongest preclinical support because it was investigated using porcine excisional and incisional wounds, biochemical and histological markers, HaCaT scratch assays, and zebrafish caudal-fin regeneration [42,51]. Direct wound-healing evidence was obtained mainly from cutaneous excisional and incisional wound models, while in vitro studies used keratinocyte or fibroblast scratch-wound assays as surrogate models of cellular migration and re-epithelialization. Zebrafish caudal-fin regeneration and angiogenesis assays provided supportive mechanistic evidence but were not considered direct mammalian cutaneous wound models. Fin regeneration involves wound-epidermis and blastema formation, followed by restoration of complex tissues, including skeletal structures. In contrast, mammalian skin repair involves haemostasis, inflammation, re-epithelialisation, granulation, extracellular-matrix deposition, contraction, and remodelling. Thus, caudal-fin findings provide supportive mechanistic evidence and require validation in mammalian wound models before translational conclusions can be drawn. *S. columbaria* and *C. orbiculata* showed promising angiogenic, fibroblast-proliferative, and collagen-related effects, but these findings remain preclinical [52]. Other Bulbine species demonstrated keratinocyte migration and caudal-fin regeneration; however, their evidence remains preliminary and requires confirmation in mammalian cutaneous wound models. Overall, the available studies largely represent acute injury or surrogate cellular models and should not be extrapolated to chronic or complex wounds, including diabetic ulcers, venous leg ulcers, pressure injuries, infected wounds, or burns, without validation in appropriate disease-specific models.

The methodological appraisal demonstrated considerable variability in study quality and reporting. In the in vivo studies, baseline comparability, outcome reporting, and completeness of outcome data were generally described more clearly than sequence generation, allocation concealment, random housing, random outcome selection, and blinding. The latter domains were frequently judged as unclear because the relevant procedures were insufficiently reported. The in vitro evidence was similarly affected by inconsistent reporting of extract standardization, concentration selection, controls, replication, and assay-specific quality safeguards. Although the included studies provided preliminary pharmacological support for several medicinal taxa, incomplete reporting and potential risks of selection, performance, and detection bias reduced confidence in the robustness and reproducibility of the overall evidence.

#### 2.2.2. Anti-Inflammatory Activity of Ethnobotanical Plants Used for Wound Healing

The anti-inflammatory evidence was derived mainly from macrophage nitric oxide assays, COX/LOX enzyme-inhibition assays, and carrageenan-, histamine-, or formalin-induced paw-oedema models. These models assess inflammatory mechanisms relevant to wound repair but do not represent open skin wounds. As the objective of this review was to evaluate the pharmacological basis underlying the traditional use of medicinal plants for wound management, it was necessary to consider biological activities that contribute to the different phases of tissue repair, particularly for species lacking direct experimental evidence of wound-healing efficacy. Anti-inflammatory activity was therefore included as a relevant pharmacological parameter because inflammation represents a tightly regulated phase of wound healing that influences the progression of subsequent reparative events. Several taxa from the ethnobotanical inventory have been evaluated in peer-reviewed South African studies for anti-inflammatory activity relevant to wound repair (Table 3). Of the 173 accepted taxa in the harmonized ethnobotanical inventory, 28 taxa (16.2%) had supportive anti-inflammatory evidence relevant to wound repair. The included studies primarily used in vitro assays, such as nitric oxide inhibition in RAW 264.7 macrophages, cyclooxygenase (COX) inhibition, lipoxygenase (LOX or 15-LOX) inhibition, and antioxidant-linked anti-inflammatory screening, together with selected in vivo models, including carrageenan-, histamine-, or formalin-induced paw oedema. These models assess inflammatory mechanisms that may be relevant to wound repair but do not directly represent open cutaneous wounds. Representative taxa include *A. aristata* (syn. *A. aristata*), *A. arborescens*, *A. ferox*, *A. afra*, *B. frutescens*, *C. edulis*, *C. orbiculata*, *E. elephantina*, *E. caffra*, *E. autumnalis*, *G. perpensa*, *L. leonurus*, *R. communis*, *S. columbaria*, and *T. capensis*. These findings are pharmacologically relevant because prolonged inflammation can delay wound repair; however, anti-inflammatory activity alone should be interpreted as supportive evidence rather than direct proof of wound-healing efficacy.

The anti-inflammatory findings presented in Table 3 should be interpreted cautiously. Assays such as NO inhibition in LPS-stimulated RAW 264.7 macrophages evaluate isolated macrophage inflammatory responses under simplified in vitro conditions and do not reproduce the complex chronic-wound microenvironment [54,55]. They do not account for interactions among keratinocytes, fibroblasts, endothelial cells, immune-cell phenotypes, extracellular matrix, impaired vascularization, persistent infection, or biofilms [56]. Furthermore, suppression of NO or inflammatory enzymes does not establish improved re-epithelialisation, granulation-tissue formation, angiogenesis, collagen remodelling, or wound closure [57]. These assays therefore provide preliminary mechanistic support only and require validation using cytotoxicity-controlled, skin-relevant, infected, and chronic-wound models before wound-healing activity can be inferred.

**Table 3:** Anti-inflammatory activity studies of medicinal plants from the ethnobotanical inventory with peer-reviewed South African evidence relevant to wound healing.

No./Species	Plant Part(s) Used	Model/Assay	Primary Citation(s)
1. *A. arborescens*	L	LOX, Nitric oxide (RAW 264.7) inhibition (in vitro)	[53]
2. *A. afra*	R	15-LOX/antioxidant (in vitro)	[58,59]
3. *A. ferox*	L	COX-1/COX-2 inhibition (in vitro); carrageenan/histamine/formalin paw oedema (rats); NO inhibition (RAW 264.7)	[53,60,61]
4. *A. marlothii*	L	COX-1/COX-2 inhibition (in vitro)	[62]
5. *A. aristata*	L	LOX, Nitric oxide (RAW 264.7) inhibition (in vitro)	[53]
6. *B. frutescens*	L; R	LOX, Nitric oxide (RAW 264.7) inhibition (in vitro)	[53]
7. *C. edulis*	L	LOX/NO screens (in vitro)	[61,63]
8. *C. orbiculate*	L	LOX/NO screens (in vitro); carrageenan paw oedema (rat)	[58,63]
9. *D. cinerea*	L	15-LOX inhibition	[64]
10. *E. autumnalis*	R; BB	15-LOX inhibition (in vitro; LOX inhibition panel)	[64]
11. *E. caffra*	BK and L	COX inhibition (in vitro)	[61]
12. *E. elephantina*	L	15-LOX inhibition (in vitro)	[65]
13. *E. capensis*	L	15-LOX inhibition	[64]
14. *G. perpensa*	R	15-LOX/antioxidant (in vitro); antinociceptive/anti-inflammatory (in vivo)	[58,63]
15. *G. fruticosus*	L	15-LOX inhibition; NO inhibition (RAW 264.7)	[63]
16. *H. caffrum*	L	15-LOX inhibition	[64]
17. *J. curcas*	L	15-LOX inhibition, NO inhibition (RAW 264.7)	[66]
18. *J. zeyheri*	R	COX-1/COX-2 inhibition (in vitro)	[62]
19. *A. oppositifolia*	L	15-LOX inhibition	[64]
20. *L. Leonurus*	L	Carrageenan/histamine paw oedema; writhing (rat)	[65]
21. *L. javanica*	L	LOX/NO inhibition (RAW 264.7)	[66]
22. *Melianthus comosus*	L	15-LOX inhibition	[64]
23. *R. communis*	L, S	NO inhibition (RAW 264.7); ABTS; COX-1/COX-2 inhibition (in vitro)	[62,67]
24. *S. columbaria*	WP	NO inhibition; zebrafish	[53]
25. *S. birrea*	L	15-LOX inhibition	[64]
26. *T. capensis*	L	15-LOX inhibition; NO inhibition (RAW 264.7)	[52,63]
27. *T. violacea*	L	15-LOX inhibition	[64]
28. *Z. mucronate*	B	COX-1/COX-2 inhibition (in vitro)	[62]

L = leaf; R = roots; BK = bark; BB = bulb; WP = whole plant.

### 2.3. Safety Profiling of Wound-Healing Medicinal Plants

Safety evidence remains substantially narrower than ethnobotanical records, and robust data supporting safe translational advancement are still limited for many South African wound-healing taxa [52]. Within the directly studied Bulbine species, available evidence indicates that several extracts were non-cytotoxic to keratinocytes at tested concentrations. However, B. latifolia showed toxicity in the zebrafish larvae model and requires more careful dose-range evaluation [51]. *Bulbine frutescens*, one of the most extensively investigated taxa in this context, has demonstrated wound-healing activity in vitro and in vivo without prominent cytotoxicity signals in the fibroblast and keratinocyte assays reported in earlier work [42,51]. Similarly, *S. columbaria* and *C. orbiculata* exhibited promising wound-healing activity with negligible in vitro toxicity in primary screening assays, and subsequent work on *C. orbiculata* further supported the low cytotoxicity profile at concentrations associated with wound-healing effects [52]. Nonetheless, ethnomedical use should not be interpreted as evidence of inherent safety, particularly for taxa such as *D. stramonium* and *J. curcas*, which are medicinally relevant but are known toxic species [65,67]. A particularly important safety concern relates to *A. oppositifolia*, a cardiotoxic species containing potent cardenolide cardiac glycosides. All parts of the *A. oppositifolia* have been reported to contain toxic cardiac glycosides, including acovenoside A and other cardioactive constituents structurally related to ouabain [68]. Given its cardiotoxic constituents and the potential for systemic exposure through disrupted skin, *A. oppositifolia* should not be recommended for empirical topical application to open or bleeding wounds in the absence of adequate dermal-safety and toxicokinetic data. This caution also extends to latex-bearing Euphorbia species, whose sap is recognized as irritating and toxic. Recent evidence on *S. africana* demonstrated moderate skin-irritation potential for the ethanol stem-bark extracts [69,70]. Efficacy-driven wound-healing research should, therefore, be paired with systematic toxicological evaluation, including cytotoxicity, dermal irritation, sensitization, repeated-dose dermal toxicity, and therapeutic-margin assessment [71,72,73].

Previous in vitro studies of *B. frutescens* reported no detectable cytotoxicity in fibroblasts and keratinocytes under the tested conditions, while in vivo studies demonstrated favourable effects on wound closure, collagen deposition, and tissue repair. However, these studies did not provide comparable efficacy and toxicity dose–response data from which a conventional therapeutic index (TI = TD_50_/ED_50_) or clinically relevant safety margin could be established. Candidate-specific evidence concerning repeated-dose dermal toxicity, cumulative irritation, skin sensitisation, dermal absorption, phototoxicity, systemic exposure, and formulation-dependent effects also remains limited. Accordingly, *B. frutescens* should be regarded as a promising preclinical wound-healing candidate with an incompletely characterised safety margin rather than as a candidate with established topical safety.

### 2.4. Botanical Conservation Status Ethnobotanically Used Wound Healing Medicinal Plants

According to the SANBI-based assessment summarized in Table 1, 144 of the 173 taxa (83.2%) were classified as least concern, while eight taxa (4.6%) were assigned categories indicating elevated conservation concern: one critically endangered, two endangered, one vulnerable, and four near threatened. The remaining 21 taxa (12.1%) were classified as not evaluated. These patterns highlight the need for conservation to not be treated as a secondary issue in medicinal plant research. The harvesting pattern recorded in this review is also ecologically informative: on a taxon basis, 44 taxa involve roots, bulbs, corms, or rhizomes, and 16 taxa involve bark. These practices may damage or kill plants if harvesting is intensive or poorly managed. These quantitative harvesting patterns are ecologically significant because the use of roots, bulbs, corms, rhizomes, and bark is inherently more destructive than leaf-based harvesting and may, therefore, increase pressure on taxa that already face conservation concern or insufficient population assessment. Particularly sensitive examples include *M. plumbea* (syn. *S. natalensis*) [74], *Al. amatymbica* [75], *B. montana, C. dentata*, and *P. africana* [76]. Species recorded as ‘Not Evaluated’ should not be assumed to be secure. Rather, they represent an additional uncertainty in conservation planning. Therefore, sustainable harvesting, cultivation, propagation, and substitution of destructive plant parts with leaves or other renewable material—when pharmacologically justified—should be prioritized.

#### Regulatory Considerations for the Collection and Use of Medicinal Plants in Wound Healing

The collection, possession, transport, storage, research use, and commercial utilization of medicinal plant material for wound healing in South Africa may be subject to different regulatory requirements depending on the species, conservation status, collection locality, source of the material, nature of the activity, and intended use. The National Environmental Management: Biodiversity Act 10 of 2004 (NEMBA) provides the principal national framework for biodiversity conservation and management, species and ecosystem protection, sustainable use of indigenous biological resources, and fair and equitable benefit-sharing arising from bioprospecting involving indigenous biological resources [77]. Activities involving listed threatened or protected species may require authorization under the applicable Threatened or Protected Species (TOPS) Regulations and associated permitting system [78]. Therefore, confirmation of the taxonomic identity and conservation status of a species should be accompanied by case-specific verification of all applicable permits and permissions before collection, transport, storage, or research use of medicinal plant material.

### 2.5. Proposed Translational Prioritization Framework

Based on the evidence synthesized in this review, Figure 2 presents a structured, context-specific organization of established best practices for the translational evaluation of South African wound-healing botanicals. The workflow progresses through taxonomic verification, ethnobotanical prioritization, standardized extract or formulation preparation, wound-phase-relevant in vitro screening, biofilm-relevant antimicrobial testing, in vivo efficacy confirmation, dermal safety assessment, and sustainability appraisal. The framework is not intended to constitute a new drug-discovery methodology. Rather, it consolidates recognised stages into a practical sequence that may assist researchers in evaluating evidence strength, identifying knowledge gaps, and avoiding the premature advancement of taxa with unresolved safety or conservation concerns.

### 2.6. Conservation–Pharmacology Mapping

Among the 173 wound-healing taxa included in the review, 145 taxa (83.2%) were classified as least concern (LC), whereas seven taxa (4.6%) fell within IUCN categories indicative of elevated conservation concern. These comprised one critically endangered species, *B. zanguebarica;* two endangered species, *A. amatymbica* and *B. montana*; one vulnerable species, *P. africana*; and four near-threatened species, *C. dentata*, *E. bicolor*, *D. delagoensis* and *M. plumbea* [Table 1]. The remaining 21 taxa (12.1%) were classified as not evaluated. Pharmacological validation of the reviewed taxa was comparatively limited. Only seven taxa (4.0%) had direct experimental evidence supporting wound-healing activity, while 28 taxa (16.2%) had indirect or supportive evidence based primarily on anti-inflammatory activity, a pharmacological property relevant to the wound-repair process. Cross-comparison of conservation status with pharmacological evidence revealed limited concordance between conservation concern and the extent of direct experimental validation. Most taxa for which direct wound-healing activity had been experimentally investigated, including *B. frutescens*, *C. orbiculata*, and *S. columbaria*, were classified as least concern.

## 3. Discussion

This review synthesizes ethnobotanical, ethnopharmacological, safety, and conservation evidence for medicinal plants used in wound care in South Africa. The current state of the field is characterized by a broad and well-documented ethnobotanical record but a comparatively narrow ethnopharmacological evidence base, with direct wound-healing validation still limited to a small subset of taxa and no South African clinical studies identified. The overall pattern is clear: traditional knowledge is extensive, but experimental validation remains limited and uneven [33,46,79]. After taxonomic harmonization, 173 accepted taxa are represented in ethnomedical practice, yet only seven taxa (4%) have been directly evaluated in wound closure assays in South Africa and 28 (16.2%) have wound-relevant anti-inflammatory evidence (Table 2 and Table 3). Accordingly, 166 taxa (96.0%) lacked direct wound-closure validation within the defined evidence scope, and no eligible South African clinical studies were identified. This pattern indicates that the literature remains predominantly ethnobotanical and preclinical. Pharmacological potential should be evaluated alongside ecological sustainability, as 42 taxa involved underground-part harvesting and 17 involved bark harvesting. The evidence map distinguishes promising taxa from those requiring conservation, safety, or regulatory safeguards before development. This imbalance explains why the field remains stronger descriptively than translationally into evidence-based use and justifies the introduction of an explicit translational prioritization framework.

A key contribution of this review is the proposal of a conservation and safety-aware translational prioritization model for South African wound-healing botanicals. Rather than ranking taxa only by historical use or isolated bioassay activity, future prioritization should integrate five evidence domains: ethnobotanical support, direct wound closure evidence, toxicological and dermal safety evidence, harvesting sustainability, and formulation feasibility. On this basis, taxa may be grouped into translational tiers. Tier 1 candidates are species with direct wound closure evidence in complementary models and an acceptable early safety signal; in the present evidence base, *B. frutescens* is the clearest example because it has been evaluated in porcine wound models, cell-based migration assays, and zebrafish regeneration assays [42,51]. Tier 2 candidates are taxa with promising wound-relevant mechanistic support but less extensive validation, including *S. columbaria* and *C. orbiculata*, which demonstrated pro-angiogenic, fibroblast-supportive, and collagen-related effects [52]. Tier 3 candidates are species supported primarily by anti-inflammatory evidence and therefore require direct wound-specific mechanistic and in vivo confirmation before translation.

Phytochemical evidence also requires more analytical integration. The available literature suggests that wound-relevant activity in southern African medicinal plants may involve compound classes such as flavonoids, triterpenes, and alkaloids [27,80]. However, the studies included in this review were too heterogeneous to support firm chemotaxonomic conclusions across all 173 taxa. Therefore, future work should combine bioactivity-guided fractionation, metabolomic profiling, and standardized extract characterization with wound-phase-specific assays. This would allow researchers to distinguish whether observed effects are associated with anti-inflammatory, antioxidant, pro-migratory, pro-angiogenic, collagen-modulating, antimicrobial, or multifunctional phytochemical profiles. This framework also helps to define what innovation should mean in this field. Innovation should not be limited to identifying additional active species; it should involve moving promising taxa from descriptive ethnobotanical relevance towards translational readiness. Accordingly, future studies should align plant selection with specific phases of wound repair, including inflammation control, re-epithelialization, fibroblast proliferation, collagen deposition, angiogenesis, infection control, and tissue remodeling. Such a phase-oriented approach would allow investigators to distinguish whether a plant acts primarily as an anti-inflammatory adjunct, a pro-regenerative candidate, an antimicrobial wound-support agent, or a multifunctional therapeutic lead.

In an investigation of Bulbine species, reported compounds include quercetin, kaempferol and its glycosides, knipholone and related phenylanthraquinones, gaboroquinones, and aloe-emodin [51]. These compounds may contribute to wound repair through antioxidants, anti-inflammatory, and antimicrobial activities, while other constituents, including saponins, terpenoids, luteolin, and apigenin, may influence collagen-related responses and inflammatory regulation. For *C. orbiculata*, saponins, flavonoids, terpenoids, alkaloids, anthraquinones, and tannins have been associated with antioxidants, antifungal, and anti-inflammatory activities [32]. In *S. columbaria*, iridoid glycosides, phenolic acids, flavonoids, and saponins have been reported, with extracts demonstrating antioxidant, nitric oxide-inhibitory, and antimicrobial activities [81]. Importantly, most available evidence is based on crude or partially characterized extracts rather than purified compounds.

Safety is a central translational issue that deserves the same emphasis as efficacy. Within the evidence assembled here, safety data for promising taxa are often limited to cytotoxicity screens, zebrafish tolerance assays, skin-irritation observations, or broader toxicological caution from non-wound contexts. Some Bulbine species extracts showed comparatively favorable safety windows in cell-based or zebrafish assays, whereas *B. latifolia* displayed toxicity at higher concentrations [51,80]. *Spirostachys africana* has also been linked to skin-irritation concerns in the current review [82]. In addition, several ethnobotanically recorded taxa such as *D. stramonium*, *J. curcas* and Euphorbia species require explicit toxicological caution when considered for topical development [83,84,85]. Although several of these plants have a history of traditional use for wound treatment, traditional use alone does not establish safety. Plants may contain phytoconstituents with known toxicological effects, and their concentration, route of exposure, and duration of use may influence the risk of adverse effects [84]. Reported or potential concerns include concentration-dependent cytotoxicity, local irritation, hypersensitivity, and systemic toxicity. For example, *B. latifolia* has shown toxicity at higher concentrations in a zebrafish model, while *D. stramonium* and *J. curcas* are recognized toxic plants. Latex-containing Euphorbia species may cause skin and ocular irritation, and *S. africana* has also been associated with irritant or toxic effects [83,86]. These findings highlight the importance of considering the toxicological profile of individual phytoconstituents when evaluating plants for topical wound applications. Therefore, future translational studies should move beyond preliminary tolerance screening and establish structured dermal toxicology pipelines that evaluate irritation, sensitization, concentration-dependent cytotoxicity, and therapeutic margins in parallel with efficacy endpoints [69,70,71,72,73,82].

Translation of ethnobotanical evidence is also limited by the poor ethnopharmacological resolution of traditional-use parameters. Many primary ethnobotanical studies do not report ethnobotanical dose, treatment frequency, duration of application, formulation details, or standardization of plant material, thereby limiting reproducibility and weakening translational interpretation [46]. A recent ethnopharmacological wound-care survey demonstrated the value in documentation of plant parts, preparation methods, application modes, and wound-stage relevance, and this level of detail should become standard in future South African studies [87]. For this reason, innovation in future work should include the reconstruction of relevant ethnobotanical preparations rather than reliance on arbitrary solvent extraction alone. Comparative testing of traditional preparations against standardized extracts, benchmark wound-care comparators, and biofilm-relevant infection models would substantially improve translational value.

The conservation dataset assembled in this review further supports a distinctive innovation pathway. Since many medicinal plants are collected for parts such as roots, bulbs, corms, rhizomes, or bark, development strategies should prioritize sustainable sourcing from the outset rather than as a downstream consideration. South African conservation research emphasizes that the sustainable utilization of medicinal plant resources must incorporate both responsible harvesting practices and the domestication or cultivation of high-demand species. Given that a substantial proportion of medicinal plants are still collected directly from wild populations, continued dependence on natural ecosystems increases the risk of resource depletion and biodiversity loss [88]. Consequently, conservation prioritization based on the South African National Biodiversity Institute (SANBI) Red List provides a scientifically robust framework for identifying species vulnerable to overexploitation and informing sustainable management strategies. A particularly useful step would be to test whether leaf-based materials or cultivated substitutes can reproduce the bioactivity of destructively harvested organs in threatened or sensitive taxa. The practical application of conservation-oriented substitution strategies is exemplified by *Warburgia salutaris*, where the use of leaves has been proposed as an alternative to the traditionally harvested bark and roots [89]. This approach aims to reduce destructive harvesting practices that threaten natural populations while maintaining access to the species for medicinal purposes. In this way, future research can move beyond efficacy screening to conservation-aware innovation that links wound-healing pharmacology with biodiversity stewardship.

Comparison of the conservation and pharmacological evidence showed limited correspondence between conservation concern and the extent of direct experimental validation. Most taxa subjected to direct wound-healing investigation, including *B. frutescens*, were not among those facing the greatest identified conservation pressure. Thus, the taxa under the greatest identified conservation pressure were not generally those supported by the strongest direct pharmacological evidence [42,51]. This finding should not be interpreted as indicating that conservation-sensitive taxa necessarily have low therapeutic potential, but rather that conservation importance and pharmacological validation represent distinct and complementary dimensions of translational prioritization. Candidate selection should therefore integrate efficacy, dermal safety, conservation status, harvesting sustainability, and the feasibility of cultivation, renewable-part use, or substitution.

The relevance of ATM extends beyond South Africa. When rigorously validated, medicinal plants used locally for wound care may contribute globally to affordable topical therapies, lead-compound discovery, and complementary wound-management strategies, especially in settings where access to advanced wound products is limited. However, translational progress requires more than positive bioassays; it also depends on quality assurance, reproducible extract preparation, regulatory oversight, and careful stewardship of indigenous knowledge. Overall, the most impactful future studies will be those that combine ethnobotanical fidelity, phytochemical standardization, wound-phase-specific bioassays, integrated safety testing, and conservation-aware sourcing in a single development pipeline.

### Strengths and Limitations

The principal strength of this review is its South Africa-specific integration of ethnobotanical documentation with wound-healing, safety, and conservation evidence in a single framework. An additional strength is the proposal of a translational prioritization model that can help guide future selection of candidate taxa for pharmacological and formulation development. However, the conclusions remain constrained by the limited number of directly validated taxa; heterogeneity in experimental models and endpoints; incomplete reporting of dose, preparation methods, and treatment regimens in primary ethnobotanical sources; and the predominance of preclinical rather than clinical evidence. These limitations should be considered when applying the prioritization framework to downstream development.

## 4. Materials and Methods

### 4.1. Database Search Strategy

#### 4.1.1. Ethnobotany Survey

A comprehensive literature search was conducted in PubMed, ScienceDirect, Web of Science, Google Scholar, and SciFinder using predefined keywords and phrases, including ethnobotanical survey, African traditional medicine, medicinal plants, wounds, wound healing, wound management, South Africa, and traditional use, applied individually and in combination. Studies were eligible for inclusion if they were peer-reviewed articles published between January 1999 and June 2026, written in English, conducted in South Africa, and reported medicinal plants traditionally used for wound healing on the basis of ethnobotanical surveys or related field documentation. Given that the scope of this review was limited to medicinal plant use within South African wound-care practice, all taxa reported in South African ethnobotanical studies were included irrespective of their biogeographical status (indigenous, naturalized, cultivated, or introduced), provided that their medicinal use was documented in South Africa and that valid binomial nomenclature was reported. Review articles, books, theses, newspaper reports, unpublished materials, animal products, and studies lacking reliable scientific plant identification were excluded from the review.

#### 4.1.2. Ethnopharmacology Evidence

Using the ethnobotanical inventory generated in Section 2.1 a secondary literature search was conducted in the same databases to identify peer-reviewed evidence on wound closure and wound-relevant anti-inflammatory activity for each recorded taxon. Search terms included “ethnopharmacology”, “traditional medicine”, “medicinal plants”, “wound healing”, “wound management”, “in vitro”, “in vivo”, “anti-inflammatory”, “cell migration”, “proliferation”, “collagen deposition”, “angiogenesis”, “wound closure”, and “South Africa”, used individually and in combination with the accepted scientific names and relevant synonyms of taxa listed in the ethnobotanical inventory. Eligible studies were peer-reviewed preclinical investigations published between January 1999 and June 2026. For direct wound closure evidence, studies were included only if they evaluated at least one wound closure endpoint, including scratch-wound closure, keratinocyte or fibroblast migration, cell proliferation, collagen deposition, angiogenesis, wound contraction, or excisional/incisional wound repair. For supportive anti-inflammatory evidence, studies were retained when taxa from the ethnobotanical inventory were assessed using recognized in vitro or in vivo anti-inflammatory models relevant to wound repair, such as inflammatory mediator inhibition, enzyme-inhibition assays, macrophage-based inflammatory models, or animal models of acute inflammation. Studies were excluded if they were not linked to taxa recorded in the ethnobotanical inventory, were conducted outside the defined South African evidence scope, or were published as conference abstracts, theses, dissertations, newspaper reports, books, or other non-peer-reviewed sources. Studies evaluating single-plant extracts, plant-derived preparations, or polyherbal formulations were eligible, whereas studies focusing solely on synthetic compounds were excluded.

#### 4.1.3. Conservation Status

The South African National Biodiversity Institute (SANBI) Red List of South African Plants was used to assign the most recent conservation category available for each taxon recorded in the ethnobotanical inventory. To strengthen the conservation appraisal, we also considered plant part(s) harvested for traditional use and checked whether legal protection or permitting requirements applied under relevant South African biodiversity and forestry regulations. Because root, bulb, corm, and bark harvesting can be destructive, these plant parts were treated as indicators of greater ecological risk than leaf-based use. Where ethnobotanical sources reported taxa under older or synonymous names, entries were harmonized under currently accepted names for counting and interpretation purposes.

### 4.2. Data Extraction, Evidence Mapping, and Synthesis

For each eligible record, data were extracted on accepted scientific name, family, plant part used, administration route, ethnobotanical source, pharmacological model, wound-relevant endpoint, safety or toxicity finding, and conservation category. Direct wound closure evidence and supportive anti-inflammatory evidence were treated as separate evidence streams to avoid overstating efficacy. The synthesis was narrative and comparative rather than meta-analytic because the included studies differed substantially in plant material, extraction method, assay system, endpoints, concentration ranges, and reporting quality. Candidate taxa were interpreted according to translational relevance, with emphasis on convergence between traditional use, wound-specific bioactivity, safety profile, and sustainability of harvested plant parts.

## 5. Conclusions

This review highlights a substantial but under-validated body of South African plant-based remedies used for wound healing. After taxonomic harmonization, 173 accepted taxa were documented ethnobotanically, but only seven taxa had direct wound closure evidence and 28 had wound-relevant anti-inflammatory support. Beyond cataloguing traditionally used taxa, this review advances a conservation- and safety-aware translational prioritization framework to guide future development of South African wound-healing botanicals. Future studies should integrate robust dose–response designs, chronic and infected wound models, biofilm-relevant assays, wound-phase-specific mechanistic endpoints, phytochemical standardization, dermal safety testing, formulation development, and sustainable sourcing strategies. Such an integrated approach is necessary to move promising ethnomedical taxa from descriptive use toward evidence-based, safe, and conservation-conscious wound therapeutics.

## Figures and Tables

**Figure 1:**
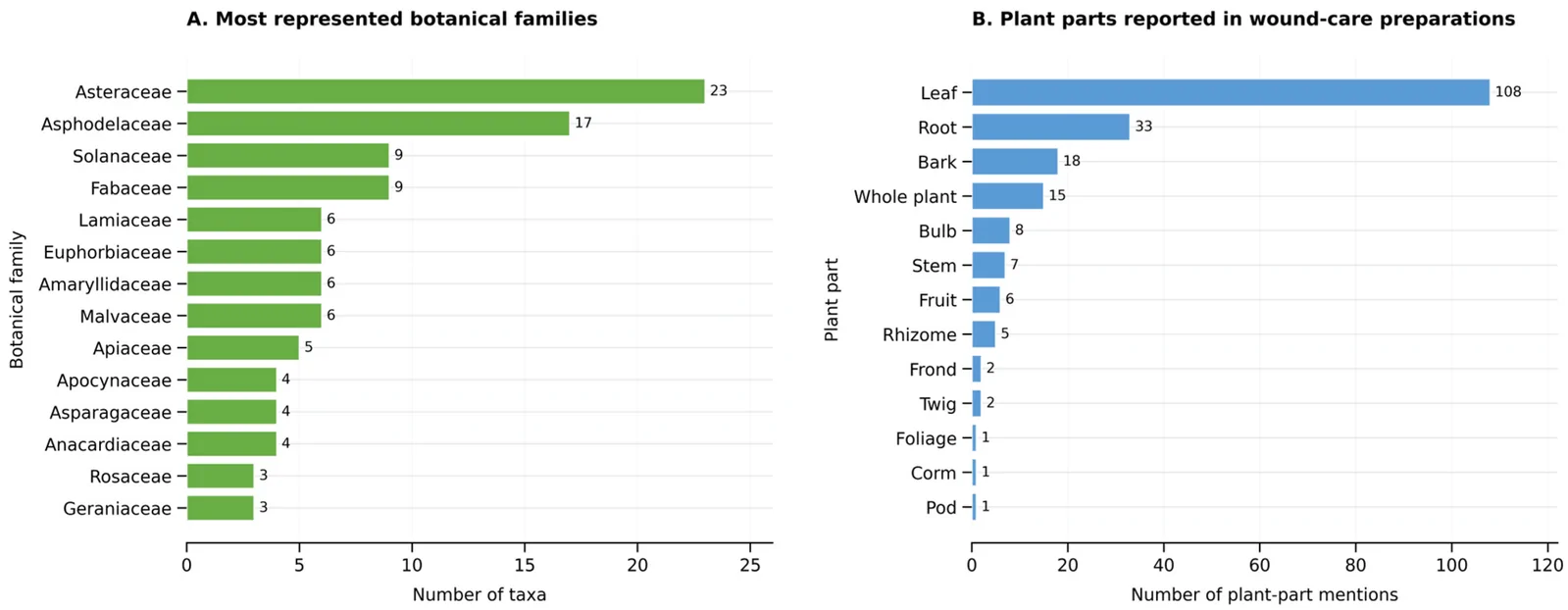
Most represented botanical families and plant parts reported for 173 medicinal plant taxa traditionally used for wound healing in South Africa.

**Figure 2:**
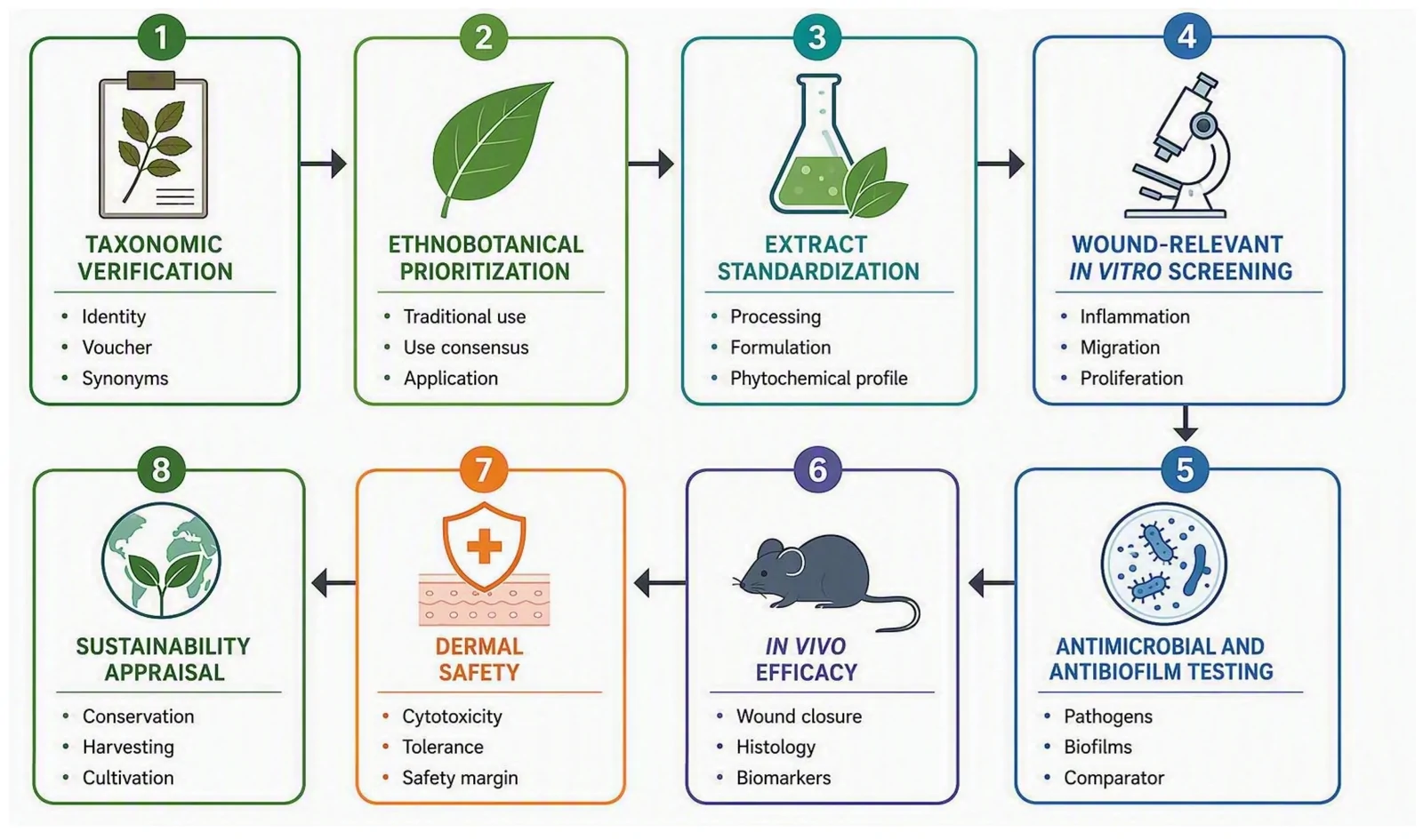
Structured best-practice workflow for the translational evaluation of South African wound-healing botanicals. The figure was designed by the authors with assistance from Microsoft 365 Copilot (Version 2606) for graphical layout and visual elements.

**Table 1:** Medicinal plants traditionally used for wound healing in South Africa after taxonomic harmonization. The taxa are further described by family, part used in traditional preparations, administration route, and the conservation status assigned using the SANBI Red List.

Family	Medicinal Plant	Part Used	Traditional Preparation	Administration Route	Conservation Status (SA)	Citation
Acanthaceae	*Barleria obtuse*	L	Paste	Topical	LC	[28]
2. Acanthaceae	*Barleria macrostegia*	R	Not reported	Paste	LC	[29]
3. Aizoaceae	*Carpobrotus edulis*	L	Juice	Topical; oral	LC	[28,30,31,32]
4. Aizoaceae	*Galenia africana*	L	Infusion	Topical	LC	[33,34]
5. Amaryllidaceae	*Boophone disticha*	BB	Paste	Topical	LC	[31,33,35,36]
6. Amaryllidaceae	*Haemanthus albiflos*	L; R	Paste, infusion	Topical; oral	LC	[28,32]
7. Amaryllidaceae	*Haemanthus coccineus*	L	Poultice	Topical	LC	[35]
8. Amaryllidaceae	*Scadoxus multiflorus*	BB	Infusion	Topical	LC	[28]
9. Amaryllidaceae	*Tulbaghia alliacea*	BB	Not reported	Topical	LC	[31]
10. Amaryllidaceae	*Tulbaghia violacea*	BB	Ash	Topical	LC	[34]
11. Anacardiaceae	*Harpephyllum caffrum*	BK	Decoction	Topical	LC	[28]
12. Anacardiaceae	*Lannea schweinfurthii*	L	Crushed	Topical	LC	[30]
13. Anacardiaceae	*Sclerocarya birrea*	B	Not reported	Topical	LC	[37,38]
14. Anacardiaceae	*Searsia lancea*	L	Poultices or paste	Topical	LC	[39]
15. Apiaceae	*Alepidea amatymbica*	R, RH	Decoction	Oral, topical	EN	[31,32]
16. Apiaceae	*Berula erecta*	WP	Not reported	Topical	LC	[33]
17. Apiaceae	*Centella asiatica*	L	Poultice	Topical	LC	[35]
18. Apiaceae	*Centella coriacea*	L	Not reported	Topical	LC	[31]
19. Apiaceae	*Heteromorpha arborescens*	L	Paste	Topical	LC	[30]
20. Apocynaceae	*Acokanthera oppositifolia*	L	Decoction	Topical	LC	[28]
21. Apocynaceae	*Carissa edulis*	L	Infusion	Topical	LC	[40]
22. Apocynaceae	*Gomphocarpus fruticosus*	WP	Not mentioned	Not reported	LC	[29]
23. Apocynaceae	*Xysmalobium undulatum*	R	Powder	Topical	LC	[32]
24. Araceae	*Zantedeschia aethiopica*	L	Dressing	Topical	LC	[28,31,35]
25. Asparagaceae	*Asparagus africanus*	L; R	Infusion, decoction	Topical; oral	LC	[28,31,32,41]
26. Asparagaceae	*Drimia capensis*	L	Not reported	Topical	LC	[41]
27. Asparagaceae	*Eucomis autumnalis*	BB, R	Decoction	Topical, roots	LC	[32,36]
28. Asparagaceae	*Merwilla plumbea* (syn. *Scilla natalensis*)	BB; R	Burned, decoction	Topical	NT	[32,34,37]
29. Asphodelaceae	*Aristaloe aristata* (syn. *Aloe aristata*)	L	Infusion	Topical	LC	[32]
30. Asphodelaceae	*Aloe aageodonta*	L	Poultice	Topical	NE	[40]
31. Asphodelaceae	*Aloe arborescens*	L	Not reported	Not reported	LC	[31]
32. Asphodelaceae	*Aloe ciliaris*	L	Not reported	Dressing	LC	[31]
33. Asphodelaceae	*Aloe ferox*	L	Decoction	Topical	LC	[28,31,32,37]
34. Asphodelaceae	*Aloe maculata*	L; S; RH	Infusino or maceration	Topical	LC	[41]
35. Asphodelaceae	*Aloe marlothii*	L	Infusion	Topical	LC	[39,40]
36. Asphodelaceae	*Aloe microstigma*	L	Lotion	Topical	LC	[34]
37. Asphodelaceae	*Aloe striatula*	L	Poultices	Poultice	LC	[35]
38. Asphodelaceae	*Aloe variegata*	L	Heated	Topical	LC	[33]
39. Asphodelaceae	*Aloe vera*	L	Leaf sap	Topical	LC	[30]
40. Asphodelaceae	*Bulbine frutescens*	L	Leaf sap	Not reported	LC	[31,42]
41. Asphodelaceae	*Bulbine lagopus*	L	Exudate	Topical	LC	[43]
42. Asphodelaceae	*Bulbine latifolia*	L	Poultice	Topical	LC	[35]
43. Asphodelaceae	*Bulbine longifolia*	L	Leaf sap	Not reported	LC	[44]
44. Asphodelaceae	*Bulbine narcissifolia*	L	Leaf sap	Not reported	LC	[44]
45. Asteraceae	*Senecio serratuloides*	L	Poultice	Topical	LC	[45]
46. Asteraceae	*Acanthospermum hispidum*	L	Mercuriation	Topical	NE	[39]
47. Asteraceae	*Acmella caulirhiza*	L	Crushed	Topical	LC	[30]
48. Asteraceae	*Arctotis arctotoides*	L	Juice	Topical	LC	[28]
49. Asteraceae	*Brachylaena discolor*	R	Infusion	Topical	LC	[28]
50. Asteraceae	*Artemisia afra*	L	Infusion	Topical	LC	[29,35]
51. Asteraceae	*Bidens pilosa*	L	Poultices, crushed	Topical	NE	[30,39]
52. Asteraceae	*Centaurea scabiosa*	WP	Maceration or poultice	Topical	NE	[41]
53. Asteraceae	*Dicoma anomala*	R	Not mentioned	Not reported	LC	[29]
54. Asteraceae	*Haplocarpha scaposa*	L	Paste	Topical	LC	[28]
55. Asteraceae	*Elytropappus rhinocerotis*	L	Infusion	Topical	LC	[43]
56. Asteraceae	*Helichrysum appendiculatum*	L	Poultice, infusion	Topical	LC	[35]
57. Asteraceae	*Helichrysum aureonitens*	L	Infusion	Topical	LC	[35]
58. Myrtaceae	*Psidium guajava*	L	Crushed	Topical	LC	[37]
59. Asteraceae	*Helichrysum foetidum*	L	Poultice	Topical	LC	[35]
60. Asteraceae	*Helichrysum nudifolium*	L	Not reported	Topical	LC	[31]
61. Asteraceae	*Helichrysum odoratissimum*	L	Infusion	Topical	LC	[28,31]
62. Asteraceae	*Helichrysum pedunculatum*	L	Juice	Topical	LC	[35]
63. Asteraceae	*Hilliardiella elaeagnoides* (syn. *Vernonia oligocephala*)	L; S	Lotion	Topical	LC	[35]
64. Asteraceae	*Senecio deltoideus*	L	Lotion	Topical	LC	[28]
65. Asteraceae	*Osteospermum herbaceum*	L	Infusion, poultice	Topical	LC	[33]
66. Asteraceae	*Calendula officinalis*	L	Lotion	Topical	NE	[45]
67. Asteraceae	*Senecio speciosus*	L	Not reported	Not reported	LC	[31]
68. Asteraceae	*Tagetes minuta*	L	Ointment	Topical	NE	[34]
69. Boraginaceae	*Lobostemon fruticosus*	L	Plaster	Topical	LC	[34]
70. Burseraceae	*Commiphora mollis*	B	Maceration	Topical	LC	[39]
71. Cactaceae	*Opuntia ficus-indica*	L	Heated	Topical	NE	[31,35]
72. Capparaceae	*Capparis tomentosa*	R	Powder	Topical	LC	[28]
73. Caprifoliaceae	*Scabiosa columbaria*	R; L	Not reported	Topical	LC	[35]
74. Apocynaceae	*Tabernaemontana elegans*	LX	Not reported	Topical	LC	[46]
75. Celastraceae	*Salacia rehmannii*	Bk	Mercuriation	Topical	LC	[39]
76. Chrysobalanaceae	*Parinari curatellifolia*	L	Decoction and poultices	Topical	LC	[39]
77. Hyacinthaceae	*Drimia delagoensis*	BB	Ash	Topical	NT	[45]
78. Crassulaceae	*Cotyledon orbiculata*	L	Poultices	Dressing	LC	[35]
79. Cucurbitaceae	*Kedrostis africana*	BB	Boiled	Topical	LC	[35]
80. Curtisiaceae	*Curtisia dentata*	BK	Infusion	Topical	NT	[35]
81. Dioscoreaceae	*Dioscorea elephantipes*	WP	Lotion, infusion	Oral, topical	LC	[32]
82. Dioscoreaceae	*Dioscorea sylvatica*	TB	Infusion	Oral	LC	[32]
83. Dryopteridaceae	*Polystichum pungens*	Fr	Powdered	Topical	LC	[35]
84. Ebenaceae	*Diospyros lycioides*	F	Juice	Topical	LC	[30]
85. Ebenaceae	*Euclea divinorum*	L	Poultices	Topical	LC	[39]
86. Euphorbiaceae	*Euphorbia bupleurifolia*	LX	Not reported	Topical	LC	[28]
87. Euphorbiaceae	*Euphorbia inaequilatera*	R	Not mentioned	Not reported	LC	[29]
88. Euphorbiaceae	*Jatropha curcas*	R	Infusion	Topical	LC	[30]
89. Euphorbiaceae	*Jatropha zeyheri*	L	Paste	Topical	LC	[30]
90. Euphorbiaceae	*Ricinus communis*	L	Poultices	Topical	NE	[35]
91. Euphorbiaceae	*Spirostachys africana*	R	Wash	Topical	LC	[39]
92. Fabaceae	*Aspalathus linearis*	WP; R	Boiled	Topical; oral	LC	[34]
93. Fabaceae	*Dichrostachys cinerea*	L, F; B, R, P	Poultice, decoction, infusion	Topical	LC	[30,39,47]
94. Fabaceae	*Erythrina lysistemon*	B	Decoction	Topical; oral	LC	[28]
95. Fabaceae	*Elephantorrhiza elephantina*	R; RH	Infusion, macerations, poultice	Topical; oral	LC	[32,41]
96. Fabaceae	*Erythrina caffra*	BK	Powdered	Topical	LC	[35]
97. Fabaceae	*Lessertia frutescens* (syn. *Sutherlandia frutescens*)	L	Infusion	Topical	LC	[48]
98. Fabaceae	*Piliostigma thonningii* (syn. *Bauhinia thonningii*)	F	Juice	Topical	LC	[30]
99. Fabaceae	*Senna occidentalis*	L	Paste, Infusion	Topical	NE	[30,35]
100. Fabaceae	*Vachellia nilotica* subsp. *kraussiana* (syn. *Acacia nilotica*)	R	Decoction	Decoction,	LC	[47]
101. Gentianaceae	*Chironia baccifera*	L	Not reported	Topical; oral	LC	[34]
102. Geraniaceae	*Pelargonium betulinum*	L	Not reported	Topical	LC	[34]
103. Geraniaceae	*Pelargonium luridum*	R	Not reported	Not reported	LC	[29]
104. Geraniaceae	*Pelargonium peltatum*	L	Poultice	Topical	LC	[35]
105. Gunneraceae	*Gunnera perpensa*	R; RH; L	Dressing	Topical	LC	[29,31,35]
106. Hyacinthaceae	*Albuca setosa*	L	Paste	Topical	LC	[28]
107. Hyacinthaceae	*Eucomis bicolor*	BB	Decoction and infusion	Oral	NT	[32]
108. Hydnoraceae	*Hydnora africana*	F	Not reported	Topical	LC	[34]
109. Hypoxidaceae	*Hypoxis hemerocallidea*	C; BB	Infussion, powder	Topical	LC	[29,35]
110. Iridaceae	*Babiana hypogaea*	L	Not reported	Not reported	LC	[29]
111. Iridaceae	*Babiana montana*	WP	Not reported	Topical	EN	[34]
112. Lamiaceae	*Acrotome inflata*	RH	Not reported	Not reported	LC	[29]
113. Lamiaceae	*Leonotis leonuris*	L, T	Infusion	Topical	LC	[35]
114. Lamiaceae	*Mentha longifolia*	L	Lotion	Topical	LC	[35]
115. Lamiaceae	*Salvia africana*	L	Burned, boiled	Topical	LC	[34]
116. Lamiaceae	*Curcuma longa*	L	Paste	Topical	LC	[45]
117. Lamiaceae	*Tetradenia riparia*	L; S	Not reported	Topical	LC	[34]
118. Lycopodiaceae	*Lycopodium clavatum*	WP	Decoction	Topical; oral	LC	[32]
119. Malvaceae	*Adansonia digitata*	B	Mercuriation	Topical	LC	[39]
120. Malvaceae	*Grewia occidentalis*	T; L	Infusion	Topical	LC	[35]
121. Malvaceae	*Hibiscus pedunculatus*	L	Burned	Topical	LC	[34]
122. Malvaceae	*Malva parviflora*	L	Paste, poultice	Topical	NE	[28,31,35]
123. Malvaceae	*Sida cordifolia*	R	Ash	Topical	LC	[30]
124. Myrtaceae	*Syzygium cordatum*	L, S	Infusion	Not reported	LC	[46]
125. Meliaceae	*Ekebergia capensis*	L	Mercuriation and poultices	Topical	LC	[35,39]
126. Meliaceae	*Trichilia emetica*	L	Poultices	Topical	LC	[31,39]
127. Melianthaceae	*Melianthus comosus*	L	Infusion	Topical	LC	[43,49]
128. Melianthaceae	*Melianthus major*	FG	Poultice	Topical	LC	[34,43]
129. Moraceae	*Chaetacme aristata*	B	Burned	Topical	LC	[46]
130. Musaceae	*Musa acuminata*	L	Not reported	Topical	LC	[30]
131. Musaceae	*Musa paradisiaca*	L	Poultices	Topical	NE	[39]
132. Nyctaginaceae	*Commicarpus pentandrus*	WP	Not reported	Not reported	LC	[29]
133. Ochnaceae	*Brackenridgea zanguebarica*	B	Maceration	Topical	CR	[39]
134. Olacaceae	*Ximenia caffra*	R	Mercuriation and poultices	Topical	NE	[39]
135. Euphorbiaceae	*Bridelia cathartica*	B	Powder	Topical	LC	[46]
136. Orobanchaceae	*Striga asiatica*	WP; R	Paste	Topical	LC	[39,49]
137. Pedaliaceae	*Harpagophytum procumbens*	WP	Not reported	Topical	NE	[34]
138. Piperaceae	*Piper capense*	Bk	Maceration	Oral, topical	LC	[39]
139. Poaceae	*Miscanthus ecklonii* (syn. *Miscanthus capensis*)	L, R	Not reported	Not reported	LC	[31]
140. Poaceae	*Zea mays*	L	Paste	Topical	NE	[28]
141. Polygonaceae	*Rumex lanceolatus*	L; S	Poultice	Topical	LC	[35]
142. Pteridaceae	*Cheilanthes hirta*	L	Not reported	Not reported	LC	[31]
143. Pteridaceae	*Cheilanthes viridis*	Fr	Powered	Topical	LC	[35]
144. Ranunculaceae	*Nigella sativa*	WP	Poultice	Topical; oral	NE	[41]
145. Rhamnaceae	*Ziziphus mucronate*	L	Poultice	Topical	LC	[39]
146. Rosaceae	*Agrimonia eupatoria*	L	Decoction	Topical	LC	[28]
147. Rosaceae	*Prunus africana*	R	Maceration	Topical	VU	[41]
148. Rosaceae	*Prunus persica*	B	Maceration	Topical	NE	[39]
149. Rubiaceae	*Coprosma robusta*	BK	Infusion	Topical	NE	[45]
150. Rubiaceae	*Pentanisia prunelloides*	L, R	Decoction, ointment	Topical	LC	[31,32,35]
151. Rutaceae	*Agathosma betulina*	L	Burned	Topical	LC	[34]
152. Rutaceae	*Coleonema album*	L	Burned	Topical	LC	[34]
153. Rutaceae	*Zanthoxylum davyi*	L; R	Crushed	Not reported	LC	[30]
154. Santalaceae	*Thesium strictum*	L	Paste	Topical	LC	[28]
155. Scrophulariaceae	*Aptosimum procumbens*	WP	Ash	Topical	LC	[33]
156. Scrophulariaceae	*Buddleja salviifolia*	BK	Infusion	Topical	LC	[35]
157. Solanaceae	*Datura stramonium*	L; S	Warmed	Topical	NE	[28,31,36]
158. Solanaceae	*Nicotiana tabacum*	L	Paste	Topical	NE	[28]
159. Solanaceae	*Physalis angulata*	L	Paste	Topical	LC	[28]
160. Solanaceae	*Solanum incanum*	R	Not reported	Topical	LC	[50]
161. Solanaceae	*Solanum lichtensteinii*	WP	Not mentioned	Not reported	LC	[29]
162. Solanaceae	*Solanum linnaeanum* (syn. *Solanum sodomeum*)	F; L	Paste	Topical	LC	[35]
163. Solanaceae	*Solanum nigrum*	L	Infusion	Topical	NE	[35]
164. Solanaceae	*Solanum panduriforme*	F	Juice	Topical	LC	[30]
165. Solanaceae	*Withania somnifera*	L, R	Poultice	Topical	LC	[31,35]
166. Sterculiaceae	*Hermannia depressa*	R	Not reported	Topical	LC	[32]
167. Thymelaeaceae	*Gnidia kraussiana*	L	Not reported	Topical	LC	[28]
168. Thymelaeaceae	*Lasiosiphon capitatus* (syn. *Gnidia capitata*)	R	Not reported	Topical	LC	[31]
169. Typhaceae	*Typha capensis*	R; S	Infusion	Topical	LC	[35]
170. Urticaceae	*Pouzolzia mixta*	L	Powder	Topical	LC	[30]
171. Urticaceae	*Urtica urens*	L	Powdered	Topical	NE	[33]
172. Velloziaceae	*Xerophyta retinervis*	R; S	Not reported	Topical	LC	[34]
173. Verbenaceae	*Lippia javanica*	L	Infusion	Topical	LC	[28]

L = leaf; R = roots; C = corms; BK = bark; BB = bulb; RH = rhizome; S = stem; WP = whole plant; T = twigs; LX = Latex; WP = whole plant; P = pods; Fr = Fronds; FG = Foliage; TB = Tuber; LC = Least Concern; NT = Near Threatened; VU = Vulnerable; EN = Endangered; CR = Critically Endangered; NE = Not Evaluated.

**Table 2:** Ethnopharmacological evidence of medicinal plants used for wound closure in South Africa.

No./Plant Species	Plant Part(s) Used	Study Type & Models	Key Findings	References	Safety/Toxicity Note
1. *B. abyssinica*	L	HaCaT scratch-wound assay (48 h); zebrafish caudal-fin regeneration	Promoted HaCaT keratinocyte migration; increased caudal-fin regeneration over time; non-cytotoxic at tested concentrations.	[51]	Non-cytotoxic at the tested concentrations reported.
2. *B. asphodeloides*	L	Keratinocyte assays; zebrafish caudal-fin regeneration	Demonstrated HaCaT keratinocyte migration; enhanced caudal-fin regeneration; safe at tested concentrations.	[51]	No marked toxicity reported at the tested concentrations.
3. *B. frutescens*	L	Porcine excisional/incisional wound model; biochemical assays; HaCaT scratch assay; zebrafish regeneration	Increased wound contraction between days 4–7; improved tensile strength and biochemical markers (protein, DNA, collagen); histology showed enhanced keratinocyte migration; promoted collagen deposition and VEGF expression; stimulated HaCaT migration and zebrafish caudal-fin regeneration.	[42,51]	Supported by multiple models; no major toxicity signal highlighted in the included wound-healing studies.
4. *B. latifolia*	L	Zebrafish toxicity assays; HaCaT scratch assay	Promoted HaCaT keratinocyte migration; increased caudal-fin regeneration; exhibited zebrafish toxicity at higher concentrations.	[51]	Zebrafish toxicity was reported at higher concentrations.
5. *B. narcissifolia*	L	Zebrafish toxicity assays; HaCaT scratch assay	Promoted HaCaT keratinocyte migration; increased caudal-fin regeneration; safe at low concentrations.	[51]	Favorable at low concentrations; broader safety margins remain to be defined.
6. *S. columbaria*	L	In vivo pro-angiogenesis in transgenic zebrafish; in vitro human dermal fibroblast (MRHF) proliferation and collagen assays	Pro-angiogenic in vivo; significantly increased fibroblast proliferation; selected extracts enhanced collagen production.	[52]	No major toxicity concern highlighted in the included assays, but dedicated dermal safety work is still needed.
7. *C. orbiculata*	L	In vivo pro-angiogenesis (zebrafish); in vitro human fibroblast (MRHF) proliferation and collagen assays	Dose-dependent pro-angiogenesis; increased fibroblast proliferation; collagen production elevated for the most active extracts.	[52]	No major toxicity concern highlighted in the included assays, but dedicated dermal safety work is still needed.

## Data Availability

All data supporting this study are contained within the article.
